# High Throughput Screening for New Fungal Polyester Hydrolyzing Enzymes

**DOI:** 10.3389/fmicb.2020.00554

**Published:** 2020-04-24

**Authors:** Simone Weinberger, Reinhard Beyer, Christoph Schüller, Joseph Strauss, Alessandro Pellis, Doris Ribitsch, Georg M. Guebitz

**Affiliations:** ^1^Department of Agrobiotechnology, Institute of Environmental Biotechnology, University of Natural Resources and Life Sciences, Vienna, Austria; ^2^Austrian Center of Industrial Biotechnology (ACIB), Tulln an der Donau, Austria; ^3^Department of Applied Genetics and Cell Biology, University of Natural Resources and Life Sciences, Vienna, Austria

**Keywords:** screening, fungi induction, polyester hydrolyzing enzymes, plastic degradation, environmentally friendly

## Abstract

There is a strong need for novel and more efficient polyester hydrolyzing enzymes in order to enable the development of more environmentally friendly plastics recycling processes allowing the closure of the carbon cycle. In this work, a high throughput system on microplate scale was used to screen a high number of fungi for their ability to produce polyester-hydrolyzing enzymes. For induction of responsible enzymes, the fungi were cultivated in presence of aliphatic and aromatic polyesters [poly(1,4-butylene adipate *co* terephthalate) (PBAT), poly(lactic acid) (PLA) and poly(1,4-butylene succinate) (PBS)], and the esterase activity in the culture supernatants was compared to the culture supernatants of fungi grown without polymers. The results indicate that the esterase activity of the culture supernatants was induced in about 10% of the tested fungi when grown with polyesters in the medium, as indicated by increased activity (to >50 mU/mL) toward the small model substrate *para*-nitrophenylbutyrate (pNPB). Incubation of these 50 active culture supernatants with different polyesters (PBAT, PLA, PBS) led to hydrolysis of at least one of the polymers according to liquid chromatography-based quantification of the hydrolysis products terephthalic acid, lactic acid and succinic acid, respectively. Interestingly, the specificities for the investigated polyesters varied among the supernatants of the different fungi.

## Introduction

Polymers and especially polyesters are components of materials with industrially interesting properties such as chemical resistance, low production costs and simple processability. Therefore, the use of plastics in food packaging, clothing, electronics, construction, and various other industrial fields resulted in a worldwide plastic production of 348 million tons in 2017 (Association of Plastics Manufacturers in Europe and European Association of Plastics Recycling and Recovery Organisations [EPRO], 2018). However, the release of petrol-based synthetic polymers into the environment poses a major threat to natural environment since they are barely biodegradable and accumulate in the ecosystems. Increased awareness of this problem led to intensive research for environmentally friendly alternatives in the last decades. One approach are biodegradable polymers, such as poly(lactic acid) (PLA) (Karamanlioglu et al., 2017), poly(butylene succinate) (PBS) (Pellis et al., 2016b) and poly(butylene adipate-*co*-terephthalate) (PBAT) (Perz et al., 2016c; Sisti et al., 2016).

Poly(butylene adipate-*co*-terephthalate) is a co-polyester consisting of the aliphatic monomers adipic acid and 1,4-butanediol and the aromatic monomer terephthalic acid (Vroman and Tighzert, 2009). Due to its special barrier properties it is widely used for food packaging or organic waste bags. Its biodegradability by microorganisms and enzymes is well known, therefore it is widely used as raw material for compostable plastics. (Perz et al., 2016b). PBAT is manufactured in industrial scale by several companies and can be used in combination with other polymers such as PLA to manufacture blended materials. To obtain PLA, mainly starches and sugars are fermented to lactic acid, which is further processed to the polymer. (Weng et al., 2013). Due to its high transparency and elastic modulus, PLA is used for disposable products and packing materials (Auras et al., 2004). The biodegradation of PLA under soil conditions is a slow process taking at least several months and depends on various factors like crystallinity and molecular weight (Shogren et al., 2003; Rudnik and Briassoulis, 2011a, b). Like PLA, PBS is another promising aliphatic biopolymer. The building blocks can be obtained *via* fermentative pathways from glucose or sucrose feedstock.

Organisms degrade polyesters by extracellular hydrolases which reduce the molar mass of the polymer to convert it to water-soluble intermediates and therefore to an accessible carbon source. Bacteria are well known producers of polyester degrading enzymes, especially of lipases and cutinases (Ronkvist et al., 2009; Ribitsch et al., 2011; Perz et al., 2016a,c). Furthermore, one of the most active enzyme on polyesters is from fungal origin, namely the cutinase from *Humicola insolens* (Weinberger et al., 2017a,b). Apart from the essential function in organisms, esterases and lipases are among the most widely used biocatalysts in the chemical industry (Wohlgemuth, 2010) and can potentially enable the recovery of the polymer building blocks. These building blocks can be used as carbon source for the fermentative production of products such as ethanol or lactic acid (Pellis et al., 2016b; Vecchiato et al., 2018). However, enzymatic hydrolysis of these polyesters is rather slow and hence more efficient enzymes would be required in order to implement an enzymatic recycling industry. In order to exploit nature for such polyester active enzymes, there is a strong demand for more efficient screening procedures which is addressed in this paper.

## Materials and Methods

### Chemicals and Reagents

Poly(1,4-butylene succinate) (PBS) powders were supplied by Goodfellow (London, United Kingdom), PBAT powders were provided by BASF (Ludwigshafen am Rhein, Deutschland). PLA powders and all other chemicals and solvents were purchased from Sigma-Aldrich at reagent grade, and used without further purification if not otherwise specified. Molecular weights [kDa] are listed in ESI Supplementary Table S2.

### Cultivation of Fungi

Fungi were cultivated in a modified Moser (1963) medium containing (per liter) 10 g casein peptone, 1 g yeast extract, 2.5 g K_2_HPO_4_, 0.25 g inositol, 375 mg CaCl_2_, 50 mg FeCl_3_, 750 mg MgSO_4_, 50 mg MnSO_4_, 5 mg ZnSO_4_, 50 g glucose, 6 g soy peptone, 0.2 g KCl, 1.44 g Na_2_HPO_4_, 0.24 g KH_2_PO_4_ and 10 g NaCl. When indicated, YES medium was also used which contains (per liter) 20 g yeast extract, 50 g sucrose, 500 mg MgSO_4_.7H_2_O, 10 mg ZnSO_4_.7H_2_O and 5 mg CuSO_4_.5H_2_O.

Fungal cultures were prepared from −80°C stocks stored as a mixture of spores and mycelium in glycerol. All isolates were isolated from environmental samples collected mainly form Central Europe and were of diverse origins ranging from soil, air and water samples to plant material or contaminated foodstuffs. Agar plates containing 33 g/L malt extract were inoculated using a sterile toothpick. Plates were then sealed and incubated in the dark at 24°C for up to 14 days or until sufficient growth and sporulation occurred. Spores were harvested by adding 10 mL sterile PBSA (Phosphate NaCl buffer) supplemented with 0.01% Tween 80 to each plate by gently scraping. The spore solution was then aspirated and stored at 4°C until further use.

A panel of 673 fungal isolates was screened for esterase activity. All pipetting steps were performed with a HAMILTON^®^ liquid handling robot (Microlab STAR) to ensure reproducibility of the technical protocol. A standardized 24-well plate format fermentation enabled us to screen fungal isolates under induced (cultivation with polymer mix) and uninduced (without polymer mix) conditions. Each fermentation was set up with 1.6 mL of the modified Moser medium supplemented with 200 μL of a mixture containing PBS, PBAT, and PLA in equal amounts (10 g/L of each polymer) suspended in 0.1 M KH_2_PO_4_ buffer (pH 7), and 200 μL spore solution. For uninduced samples 200 μL 0.1 M KH_2_PO_4_ buffer was added instead of polymer mix. Samples were incubated for 24 days at 24°C with gentle shaking. A control was added to each batch by adding 200 μL sterile PBSA instead of the spore solution. Each fermentation was run in 12 replicates and supernatants were subsequently pooled.

Upon harvest, the mycelium was manually removed using a sterile toothpick, the supernatant was then aspirated, transferred into a fresh vial and centrifuged at 3200 *g* for 20 min to remove residual mycelium. Subsequently, the supernatant was filtrated using a 0.2 μm polystyrene filter to obtain sterile filtrate and stored at −20°C until further use.

### Esterase Activity

For activity measurements of induced and uninduced supernatants, *para*-nitrophenyl butyrate (pNPB) was used as substrate. The final assay mixture consisted of 200 μL of the substrate solution (86 μL of pNPB and 1000 μL of 2-methyl-2-butanol, added to 25 mL 0.1 M KH_2_PO_4_ buffer pH 7) and 50 μL of sample. The increase of the absorbance at 405 nm due to the hydrolytic release of *p*-nitrophenol at 405 nm [ε405 nm = 9.72 (mM cm)^–1^] was measured over 10 min in cycles of 18 s at 30°C with a Synergy H1 microplate reader (BioTek Instruments, VT, United States) using 96-well micro-titer plates (Greiner 96 Well Flat Bottom Transparent Polystyrene). A negative control was included on each plate using 50 μL 0.1 M KH_2_PO_4_ buffer instead of supernatant. The activity was calculated in units (U), where 1 unit is defined as the amount of extract required to hydrolyze 1 μmol of substrate per minute under the given assay conditions.

### Polymer Hydrolysis

Polymer powders (5.0 mg) were incubated with 1 mL of the culture supernatants showing a pNPB hydrolytic activity >50 mU/mL. Incubations were conducted for 21 days in an orbital shaker set at 100 rpm and 65°C since good enzyme stability and activity over time for the hydrolysis of various polyesters had been previously reported for these conditions (Pellis et al., 2016a; Gamerith et al., 2017b; Weinberger et al., 2017b). Blank reactions were carried out in YES medium. All reactions were performed in duplicates.

### High-Performance Liquid Chromatography (HPLC)

After incubation, supernatants incubated with PBAT were diluted with ice-cold methanol (1:1 v/v) to precipitate the enzymes. The samples were centrifuged at 12,700 rpm (Centrifuge 5427 R, Eppendorf, Germany) for 15 min at 4°C, followed by a filtration through 0.45 μm nylon syringe filters into high performance liquid chromatography (HPLC) vials. For analysis of the samples, a HPLC-DAD system consisting of a 1260 Infinity (Agilent Technologies, Palo Alto, CA, United States) coupled with a reversed phase column C18 (Poroshell 120 EC-C18 2,7 μm 3.0 × 150 mm) was used. The PBAT hydrolysis products were separated using a non-linear gradient as previously described (Quartinello et al., 2017) and detected with a photodiode array detector (Agilent Technologies, 1290 Infinity II, Vienna, Austria) at the wavelength of 245 nm. For the quantification of the released products bis(4-hydroxybutyl) terephthalate (BTaB), mono(4- hydroxybutyl) terephthalate (BTa) and terephthalic acid (Ta) (BASF, Ludwigshafen am Rhein, Deutschland), was used. For the quantification of the degradation products, external calibration curves in the range 0.005-1 mM (see ESI, Supplementary Figure S1) were used. Blanks were subtracted from the results.

Hydrolysis samples with PLA and PBS were precipitated following the Carrez method (Perz et al., 2016b) and filtered through 0.20 μm Nylon filters (GVS, Indianapolis, United States). The analytes were separated by HPLC using refractive index detection (1100 series, Agilent Technologies, Palo Alto, CA, United States) equipped with an ICSep−ION−300 column (Transgenomic Organic, San Jose, CA, United States) of 300 mm by 7.8 mm and 7 μm particle diameter. Column temperature was maintained at 45°C. Samples (40 μL) were injected and separated by isocratic elution for 40 min at 0.325 mL min^–1^ in 0.005 M H_2_SO_4_ as the mobile phase. Lactic acid and succinic acid were used for the calibration. For the quantification of the degradation products, external calibration curves in the range 0.01–20 mM (see ESI, Supplementary Figure S2) were used. Blanks were subtracted from the results.

## Results and Discussion

Screening of a culture collection of environmental fungal isolates collected mainly in Central Europe was performed in a partially automated setup to increase reproducibility and speed of the technical protocols applied (see ESI, Supplementary Figure S3). Inoculation of fungi was performed on a Hamilton liquid handling robot (Hamilton Microlab STAR) while screening was conducted in a 2-stage process. First, fungi were challenged with a polymer mix containing PBS, PLA, and PBAT and supernatants were tested for esterase activity. Fungal isolates which showed an activity above a soft threshold of 50 mU/mL were selected for a further second round of screening. During this second screening, fungal isolates were additionally cultivated without polymer mix to identify polymer-inducible esterase activity.

## Inducibility

**FIGURE 1:**
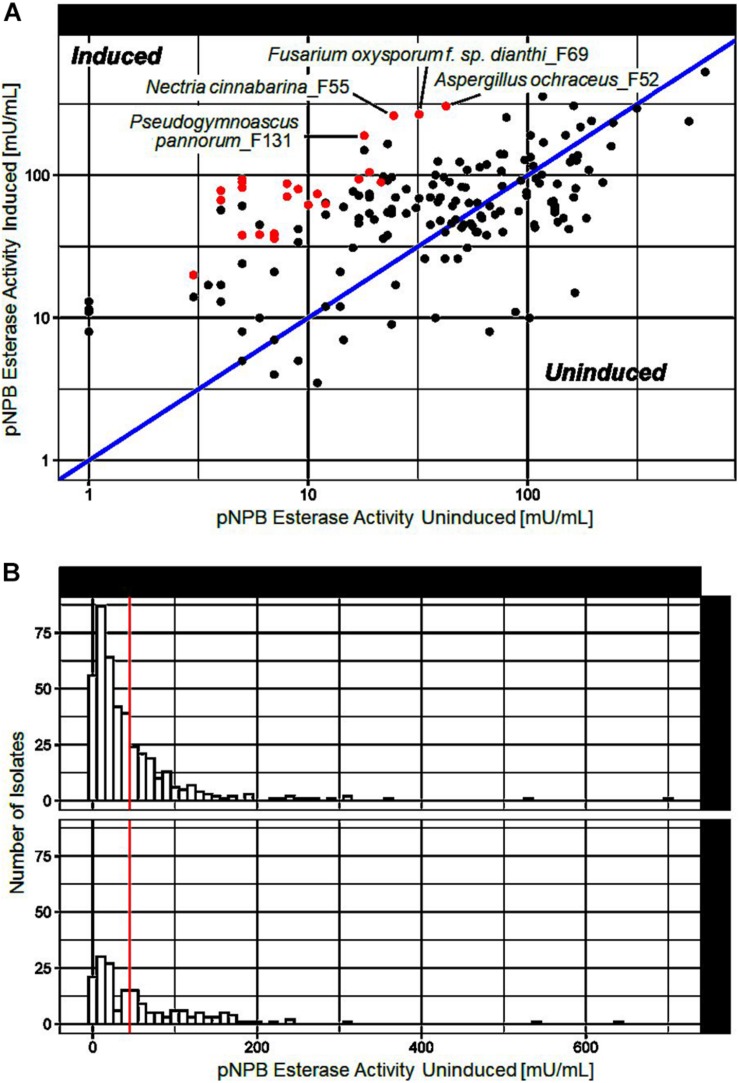
pNPB esterase activities measured after cultivation of different fungi in presence of a polyester mix (induced) or without (uninduced). **(A)** Comparison of pNPB esterase activities under induced and uninduced conditions. Each point represents a fungal isolate. Isolates above the blue line produced supernatants with higher pNPB esterase activity when co-cultivated with a polymer mix. 24 isolates marked in red were selected for further investigation. **(B)** Histogram of pNPB esterase activity of all tested fungal isolates under induced and uninduced conditions. The vertical red line indicates the arbitrary threshold of 50 mU/mL, above which pNPB esterase activity was considered substantial.

To investigate the induction of secreted polyester hydrolyzing enzymes, 673 different fungi were cultivated in the presence of a polymer mix of different aliphatic and aromatic polyesters, namely, PBS, PLA, and PBAT. After 3 weeks incubation esterase activity of the supernatants from polymer induced and control incubations were measured as described earlier. Out of the 673 fungi investigated, 66 strains showed elevated activity when cultivated in the presence of polyesters (Figure 1). Several isolates generated supernatants with substantial esterase activity (>50 mU/mL) under induced conditions and are highlighted in Figure 1A. Figure 1B shows the distribution of esterase activities measured during our screen.

**FIGURE 2:**
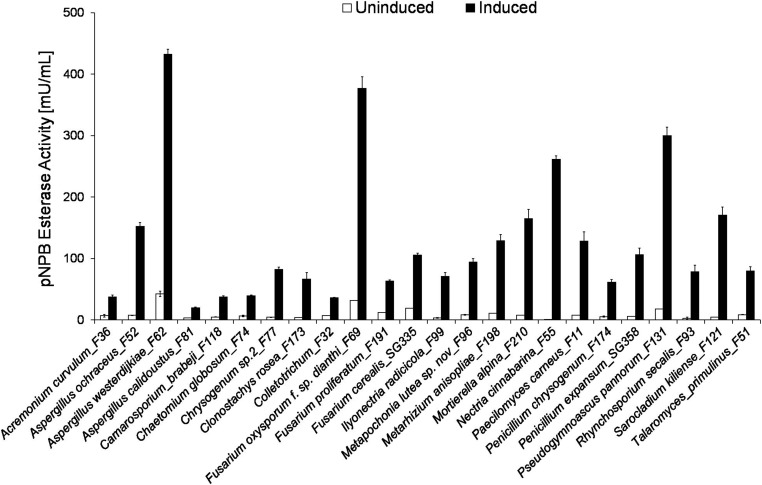
Induction of fungal extracellular pNPB esterase activity when cultivated in the presence of different aliphatic and aromatic polyesters. Comparison of pNPB esterase activity [mU/mL] in induced (gray) and uninduced (white) supernatants. All pNPB esterase measurements were conducted in triplicates with standard deviations indicated.

The 24 strains exhibiting strongest induction are shown in Figure 2. These included three representatives of *Aspergillus* sp. and *Fusarium* sp., respectively, as well as *Penicillum s*pecies, which are mold fungi and typical soil organisms. Representatives of all three genera were previously reported to produce extracellular esterases (Gautam et al., 2007; Mathur and Prasad, 2012; Hu et al., 2016; Álvarez-Barragán et al., 2016; Carniel et al., 2017; Khan et al., 2017; Osman et al., 2018). The highest induction from 1 to 261 mU/mL was found for *Nectria cinnabarina_*F55. Furthermore, *Sarocladium kiliense*_F121, *Rhynchosporium secalis*_F93, *Ilyonectria radicicola*_F99 and *Mortierella alpina_*F210 showed strong induction of extracellular esterase activity (see ESI, Supplementary Table S1).

As fungal plant pathogens, *N. cinnabarina_*F55 are well known to produce extracellular enzymes capable of degrading cell wall components such as cutin, since they are essential during the invasion of the host. This is in good agreement with our previous results indicating that cutinases hydrolyze very efficiently synthetic polyesters (Pellis et al., 2016a; Weinberger et al., 2017b; Vecchiato et al., 2018). Enzyme production by *N. cinnabarina_*F55 was studied in the presence of natural substrates or plant cells (Purdy and Kolattukudy, 1973; Eddine et al., 2001; Hawthorne et al., 2001). Eddine et al. (2001) reported an extracellular lipase from *Nectria haematococca* (anamorph *Fusarium solani* f. sp. *pisi*) which showed an esterase activity of 12 U/mL for *p*-nitrophenyl palmitate, but was not tested on polymeric substrates. However, native and engineered esterases/cutinases from *F. solani* f. sp. *pisi* have previously been reported to hydrolyze polyesters including PET [poly(ethylene terephthalate)] (Araújo et al., 2007). Bridge et al. (1989) reported already in 1989 the production of esterases by different *Sarocladium* species (anamorph *Acremonium* sp.). Furthermore, *Acremonium* sp. was identified as producer of enzymes capable of degrading cellulose and chitin (Baldrian et al., 2011). *R. secalis*_F93 and *I. radicicola*_F99 are pathogens of barely and other plants such as *Stellera chamaejasme* L, so research is focused on defending mechanisms of the host and not on the enzymes of the fungi (Daâssi et al., 2016; Marzin et al., 2016; Penselin et al., 2016; Looseley et al., 2018). Overall, there is not much information available regarding the hydrolytic enzymes derived from these organisms.

A lipase was reported from *Mortierella echinosphaera*. Esterase activity was found on p-nitrophenyl esters of different chain length (C2–C16), and potential for catalysis of polymerization reactions (Kotogán et al., 2018). Addition of lipids to the cultivation medium of *Mortierella* sp. resulted in induction of activities (Gaspar et al., 1999; Kotogán et al., 2014).

### Hydrolysis of Polyesters by Fungal Supernatants

**FIGURE 3:**
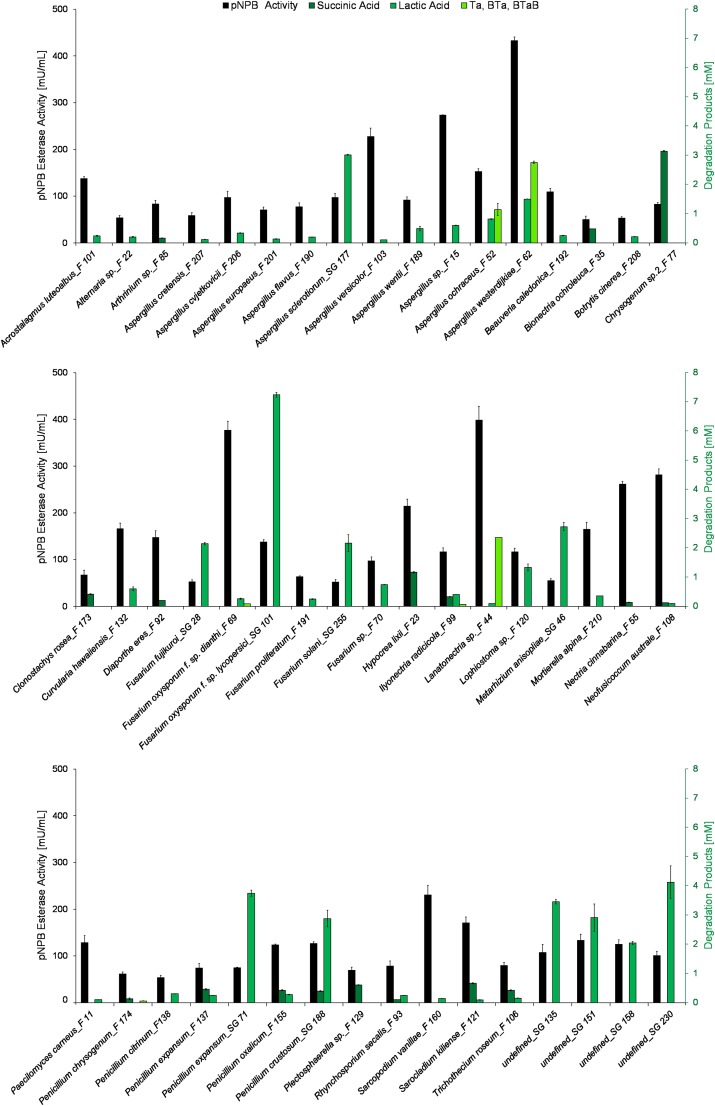
Hydrolysis of different polyesters by supernatants of fungi cultivated in the presence of aliphatic and aromatic polyesters for enzyme induction. pNPB esterase activities [mU/mL] (gray) of fungal culture supernatants were compared with esterase activities on polymers which are shown as the amount of hydrolysis products after 3 weeks of incubation. Monomers from PBS (succinic acid) are shown in dark green, PLA products (lactic acid) are shown in green while hydrolysis products from PBAT (TA, BTA, BTAB) are shown in light green. All experiments were performed in duplicates as indicated by ± standard deviation.

Those fungal supernatants showing pNPB hydrolytic activity of above 50 mU/mL were subsequently incubated with PLA, PBS, and PBAT, respectively. The quantification of the resulting hydrolysis products is shown in Figure 3. Although the pNPB esterase activity is relatively low when compared to commercial esterases such as Lipase B from *Candida antarctica* and a cutinase from *H. insolens*, several species of *Aspergillus*, *Fusarium*, and *Penicillium* were found to efficiently hydrolyze the tested polyesters (Carniel et al., 2017). Forty one of the 50 polymer-active strains degraded PLA, but to a different extent. The highest amount of lactic acid (7 mM) was produced by *Fusarium oxysporum* f. sp. *lycopersici_*SG 101 Amongst the tested polymers, the *F. oxysporum* f. sp. *lycopersici_*SG 101 supernatant showed a high activity on PLA. Interestingly, this supernatant had a relatively low activity of 138 mU/mL on pNPB, compared to *Aspergillus westerdijkiae_*F 62 (433 mU/mL), *Lanatonectria* sp._F 44 (398 mU/mL) and *Sarcopodium vanillae_*F 160 (230 mU/mL). This demonstrates that activity on the small substrate pNPB is suitable for high throughput pre-screening. However, this activity cannot be used to predict hydrolysis activity on distinct polyesters (Gamerith et al., 2017a; Weinberger et al., 2017b). Various *Fusarium* sp. are known to produce cutinases (Zhao et al., 2005; Mao et al., 2015), lipases (Shimada et al., 1993; Dell et al., 2016; Facchini et al., 2018), and other esterases (Luo et al., 2015; Ahuactzin-Pérez et al., 2016). Although both PBS and PLA are linear polyesters, only 17 of the fungi were able to degrade PBS. Seven of those strains were active only on PBS. The most active strain, a *Chrysogenum* sp. 2_F 77, produced 3 mM succinic acid. The difference between PLA and PBS is most probably caused by the different crystallinities of these two aliphatic polymers. Since PBS is characterized by higher crystallinity, the tightly packed chains of this polymer avert enzymatic attack (Cho et al., 2001; Gigli et al., 2013). *Chrysogenum* sp. are known to produce acetyl xylan esterases able to cleave acetyl substituents from acetylated xylan, an important step for the degradation of lignocellulose. Yang et al. (2017) reported the activity of an acetyl xylan esterase from *Chrysogenum* P33 with an activity on p-nitrophenyl acetate of 35 mU/mg purified enzyme.

It is not surprising that PBAT was the most difficult polymer to hydrolyze for the fungal esterases due to its partially aromatic nature. *A. westerdijkiae_*F 62 was most efficient in the hydrolysis of PBAT as judged by the formation of 2.8 mM hydrolysis products. In relation to the different activities on pNPB (short model substrate), the data show the different substrate specificities of the esterases secreted by the different fungi on PBAT, PBS, and PLA. The identification of such specificities can potentially enable the implementation of enzymes in processes related to substrate and product niches e.g., the selection of appropriate strains could be exploited for polymer degradation.

## Conclusion

Our library screening procedure resulted in the identification of several fungal strains capable of producing enzymes with hydrolytic activity on aromatic and aliphatic polyesters. This function-based search method is the first step for the identification of fungi capable to become a novel source of enzymes active on polymers. In this work we demonstrated that the incubation of fungi with the target polymers can induce the production and secretion of enzymes hydrolyzing these polymers.

A further investigation of the supernatants resulting in identification and recombinant expression of the enzymes is currently ongoing and represents the next step toward a deeper understanding of the different tools that fungi could provide for an enhanced polymer degradation that would allow the closure of the carbon cycle.

## Data Availability Statement

All datasets generated for this study are included in the article/Supplementary Material.

## Author Contributions

GG, CS, DR, SW, and RB conceived and designed the experiments. SW and RB performed the experiments and analyzed the data. AP analyzed the used polyesters. SW, GG, DR, CS, JS, and AP wrote the manuscript.

## Conflict of Interest

The authors declare that the research was conducted in the absence of any commercial or financial relationships that could be construed as a potential conflict of interest.
