# Supplementary material for: High Throughput Screening for New Fungal Polyester Hydrolyzing Enzymes
Source: Front Microbiol. 2020 Apr 24;11:554. doi: 10.3389/fmicb.2020.00554 (PMC7193820; doi:10.3389/fmicb.2020.00554)
Supplement: Supplementary file 1 [file Data_Sheet_1.docx]

High throughput screening for new fungal polyester hydrolyzing enzymes

Simone Weinberger^1, 2^, Reinhard Beyer^2^, Christoph Schueller^2^, Alessandro Pellis^1^, Doris Ribitsch^1, 2^, Georg M. Guebitz,^1, 2^

^1^ University of Natural Resources and Life Sciences, Vienna, Department of Agrobiotechnology, Institute of Environmental Biotechnology, Konrad Lorenz Strasse 20, 3430, Tulln an der Donau, Austria

^2^ University of Natural Resources and Life Sciences, Vienna, Department of Applied Genetics and Cell Biology, Konrad Lorenz Strasse 24, 3430, Tulln an der Donau, Austria

^3^ Austrian Centre of Industrial Biotechnology (ACIB), Konrad Lorenz Strasse 20, 3430, Tulln an der Donau, Austria

*** Correspondence:** Dr. Doris Ribitsch, email: [doris.ribitsch@boku.ac.at](mailto:doris.ribitsch@boku.ac.at)


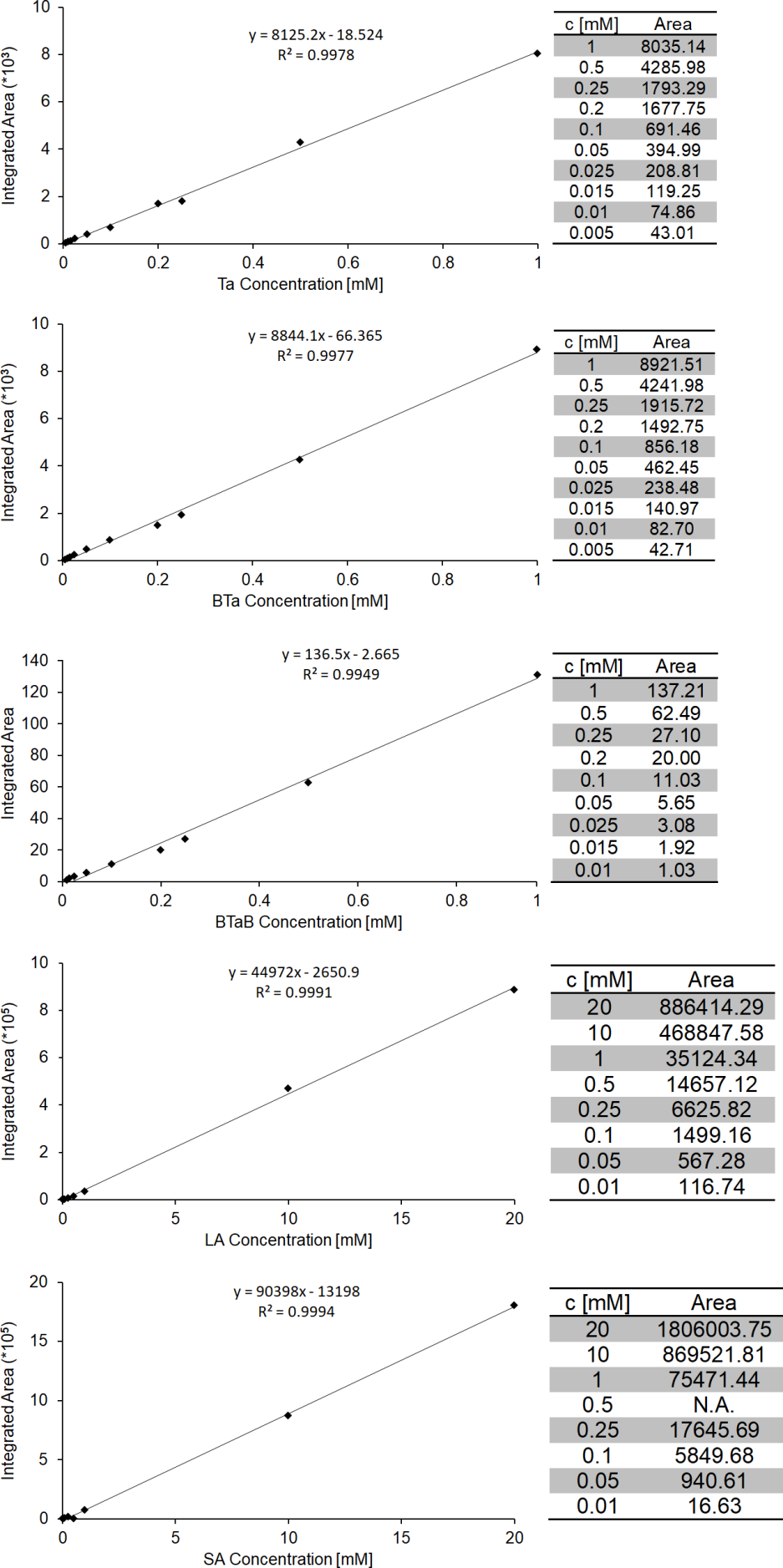


**Figure S1:** Ta, BTa and BTaB calibration used for the analysis of molecules released from PBAT by the enzymes in fungi supernatants


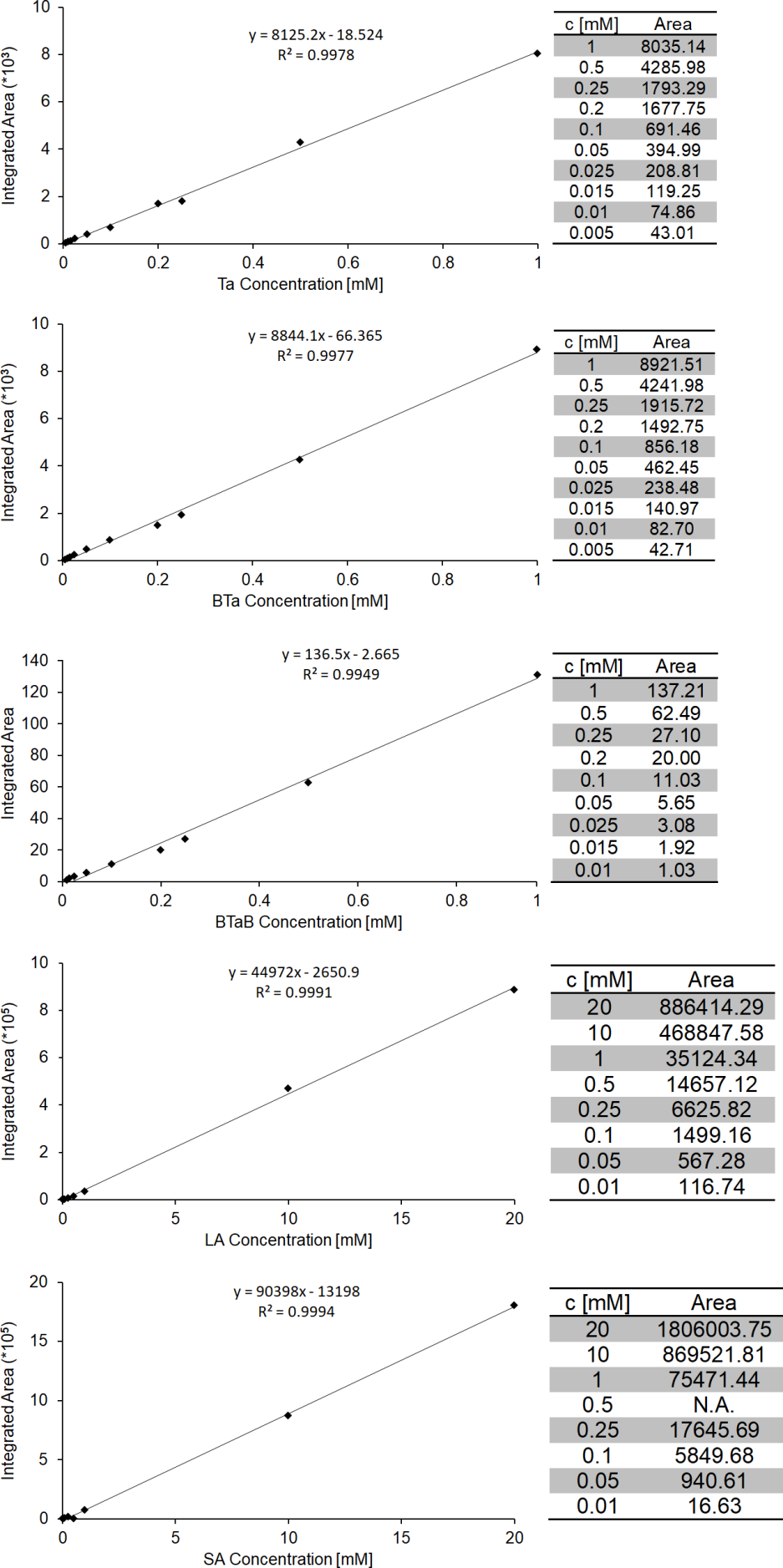


**Figure S2:** calibrations used for the analysis of LA and SA released from PLA and SA by the enzymes in fungi supernatants

**Table S1:** comparison of pNPB [mU/mL] in uninduced and induced supernatants.

| Species | uninduced | | induced | |
| --- | --- | --- | --- | --- |
|  | pNPB Activity  [mU/mL] | Stdv. | pNPB Activity  [mU/mL] | Stdv. |
| *Acremonium curvulum_F36* | 6.97 | 2.16 | 37.74 | 2.89 |
| *Aspergillus ochraceus_F52* | 7.72 | 0.40 | 152.44 | 6.44 |
| *Aspergillus westerdijkiae_F62* | 42.50 | 4.38 | 432.66 | 7.69 |
| *Aspergillus calidoustus_F81* | 3.34 | 0.22 | 19.77 | 1.46 |
| *Camarosporium_brabeji_F118* | 4.93 | 0.50 | 37.70 | 1.74 |
| *Chaetomium globosum_F74* | 6.51 | 1.29 | 39.16 | 1.31 |
| *Chrysogenum sp. 2_F77* | 4.54 | 0.27 | 82.40 | 3.85 |
| *Clonostachys rosea_F173* | 4.00 | 0.00 | 66.67 | 10.77 |
| *Colletotrichum_F32* | 7.00 | 0.00 | 36.36 | 0.71 |
| *Fusarium oxysporum f. sp. dianthi_F69* | 32.00 | 0.00 | 376.76 | 19.25 |
| *Fusarium proliferatum_F191* | 12.00 | 0.00 | 63.34 | 2.03 |
| *Fusarium cerealis_SG335* | 19.00 | 0.00 | 105.50 | 3.11 |
| *Ilyonectria radicicola_F99* | 3.38 | 0.68 | 71.06 | 6.31 |
| *Metapochonia lutea sp. nov_F96* | 8.56 | 0.86 | 94.14 | 5.65 |
| *Metarhizium anisopliae_F198* | 11.00 | 0.00 | 129.27 | 9.92 |
| *Mortierella alpina_F210* | 8.00 | 0.00 | 164.77 | 15.27 |
| *Nectria cinnabarina_F55* | 0.52 | 0.71 | 261.58 | 5.71 |
| *Paecilomyces carneus_F11* | 8.00 | 0.00 | 128.44 | 15.05 |
| *Penicillium chrysogenum_F174* | 5.44 | 0.89 | 61.56 | 4.08 |
| *Penicillium expansum_SG358* | 6.00 | 0.00 | 106.22 | 10.88 |
| *Pseudogymnoascus pannorum_F131* | 18.00 | 0.00 | 299.87 | 13.97 |
| *Rhynchosporium secalis_F93* | 2.95 | 1.90 | 78.35 | 11.20 |
| *Sarocladium kiliense_F121* | 5.00 | 0.00 | 170.56 | 13.12 |
| *Talaromyces_primulinus_F51* | 8.75 | 0.46 | 79.75 | 6.96 |

| **Table S2:** Analysis of PLA, PBS and PBAT. | | | |
| --- | --- | --- | --- |
| **Polymer** | **M_n_ [Da]** | **M_w_ [Da]** | **D [M_w_/M_n_]** |
| PLA | 549000 | 862000 | 1.6 |
| PBS | 22000 | 45000 | 2.1 |
| PBAT | 34000 | 85000 | 2.5 |
| Data calculated using a linear polystyrene calibration (266-1170000 Da) | | | |


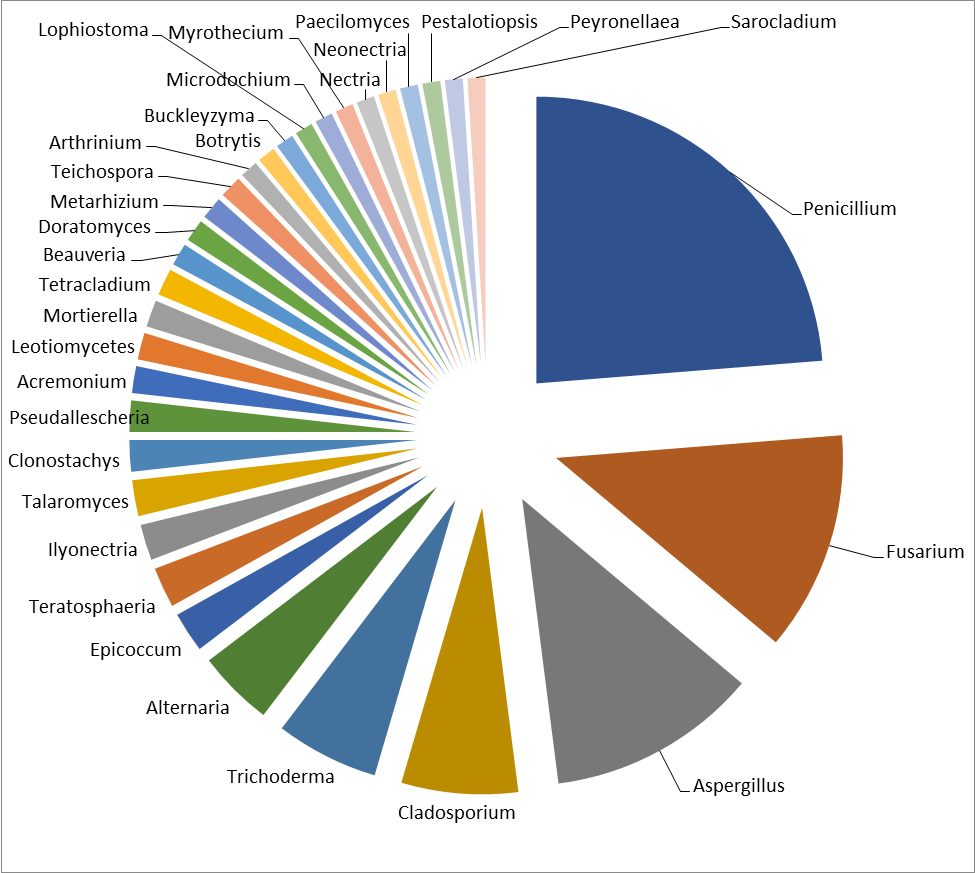


**Figure S3:** pie chart showing genera represented with more than 3 isolates included in the study. *Penicillium*, *Fusarium*, *Aspergillus*, *Cladosporium*, *Trichoderma*, *Alternaria*, and *Epicoccum* represent about 50% of the strains.
